# Temozolomide and the PARP Inhibitor Niraparib Enhance Expression of Natural Killer Group 2D Ligand ULBP1 and Gamma-Delta T Cell Cytotoxicity in Glioblastoma

**DOI:** 10.3390/cancers16162852

**Published:** 2024-08-15

**Authors:** Amber B. Jones, Kaysaw Tuy, Cyntanna C. Hawkins, Colin H. Quinn, Joelle Saad, Sam E. Gary, Elizabeth A. Beierle, Lei Ding, Kate M. Rochlin, Lawrence S. Lamb, Anita B. Hjelmeland

**Affiliations:** 1 Department of Cell, Developmental and Integrative Biology, University of Alabama at Birmingham, Birmingham, AL 35294, USA; amberj96@uab.edu (A.B.J.); ktuy@uab.edu (K.T.); cyntanna@gmail.com (C.C.H.); jsaad@uab.edu (J.S.); sgary@uab.edu (S.E.G.); 2 Medical Scientist Training Program, University of Alabama at Birmingham, Birmingham, AL 35294, USA; chquinn@uab.edu; 3 Department of Surgery, University of Alabama at Birmingham, Birmingham, AL 35294, USA; elizabeth.a.beierle@childrensal.org; 4 In8Bio, Inc., New York, NY 10118, USA; lding@in8bio.com (L.D.); kmrochlin@in8bio.com (K.M.R.); larry@in8bio.com (L.S.L.)

**Keywords:** natural killer group 2D ligands, PARP inhibition, DNA alkylating agent, gamma delta T cell, cell-based immunotherapy

## Abstract

**Simple Summary:**

The urgent need for novel therapies for glioblastoma (GBM) treatment may be met through an increased understanding of immune escape and improved immunotherapies. Immune escape can be caused by reduced expression of NKG2D ligands (NKG2DL) on tumor cells that are recognized by natural killer or cytotoxic T cells. While DNA damage from therapeutics can increase NKG2DL ligands to enable immune cell recognition, the immunosuppressive side effects of chemotherapies may limit this potential benefit, especially in the absence of immunotherapy interventions such as gamma delta T cells. PARP inhibitors sensitize cancer cells to temozolomide-induced cell death, and the combination is in clinical trials for GBM. We find that temozolomide and the PARP inhibitor, niraparib, can increase NKG2DL in GBM cells derived from multiple PDXs and increase GBM cell killing by gamma delta T cells in cells derived from a GBM PDX. We suggest capitalizing on transient chemotherapy-induced upregulation of NKG2DL by the combination of temozolomide and PARP inhibition in the appropriate immunotherapy setting for GBM treatment.

**Abstract:**

Glioblastoma (GBM) is an immunologically cold tumor, but several immunotherapy-based strategies show promise, including the administration of ex vivo expanded and activated cytotoxic gamma delta T cells. Cytotoxicity is partially mediated through interactions with natural killer group 2D ligands (NKG2DL) on tumor cells. We sought to determine whether the addition of the blood–brain barrier penetrant PARP inhibitor niraparib to the standard of care DNA alkylator temozolomide (TMZ) could upregulate NKG2DL, thereby improving immune cell recognition. Changes in viability were consistent with prior publications as there was a growth inhibitory effect of the combination of TMZ and niraparib. However, decreases in viability did not always correlate with changes in NKG2DL mRNA. *ULBP1*/*Mult-1* mRNA was increased with the combination therapy in comparison to either drug alone in two of the three cell types tested, even though viability was consistently decreased. mRNA expression correlated with protein levels and ULBP1/MULT-1 cell surface protein was significantly increased with TMZ and niraparib treatment in four of the five cell types tested. Gamma delta T cell-mediated cytotoxicity at a 10:1 effector-to-target ratio was significantly increased upon pretreatment of cells derived from a GBM PDX with TMZ and niraparib in comparison to the control or either drug alone. Together, these data demonstrate that the combination of PARP inhibition, DNA alkylation, and gamma delta T cell therapy has the potential for the treatment of GBM.

## 1. Introduction

Glioblastomas (GBM) have proven to be clinically difficult to treat because of limited and often unsuccessful cancer therapies, justifying the identification of novel treatment strategies. The current standard of care treatment for GBM includes safe maximal resection followed by adjuvant radio- and chemotherapy with the DNA alkylating agent temozolomide (TMZ) [1]. To improve the efficacy of chemotherapies, including TMZ and radiotherapy, targeting poly (ADP-ribose) polymerase (PARP)-dependent DNA repair has been and continues to be investigated [2,3,4]. PARylation of the enzyme that repairs TMZ-mediated DNA damage [O^6^-methylguanine DNA methyltransferase (MGMT)] is required for its activity, pointing to synergistic effects with TMZ and PARP inhibitor treatment [4]. Indeed, the combination of TMZ and PARP inhibition against gliomas (sometimes including irradiation) is being or has been tested in clinical trials (NCT03212742; NCT03749187; NCT03914742; NCT01514201; NCT00994071; NCT02152982).

In addition to improving chemotherapeutic regimens, GBM treatment may be enhanced through an increased understanding of immunosuppression and immune escape [5] and impacts on T lymphocytes (T cells) [6]. T cells represent the adaptive side of the immune system and are made up of several subsets. Few T cells infiltrate into the tumor microenvironment, and these are often immunosuppressive T regulatory cells (T_regs_) [7]. There are a few effector T cells that migrate towards GBMs, but they are usually dysfunctional with a lack of clonal expansion and cytotoxic function. Effector T cells play a vital role during immune surveillance as patients with competent T cells tend to fare better compared with patients with more dysfunctional T cells [7,8]. The lack of T cell infiltration and activation in the tumor microenvironment is due to immune-suppressive myeloid cells and immune checkpoints [9,10]. Immunotherapies for GBM are being developed to overcome immunosuppression and optimize effector T cell function.

One rare effector T cell population that has been used for immunotherapies against GBM in clinical trials is gamma delta T cells [11,12,13,14,15]. These clinical trials include genetically engineered, TMZ-resistant gamma delta T cells that could remain active even in the presence of TMZ (NCT04165941; NCT05664243). Gamma delta T cells produce perforin and granzyme B for direct tumor lysing or cytokines to stimulate macrophages and dendritic cells. Importantly, gamma delta T cells recognize antigens in an MHC unrestricted manner and the absence of co-simulations. Gamma delta T cells express the C-type lectin-like receptor—NKG2D, a well-studied receptor with critical roles in tumor recognition [16,17]. NKG2D recognizes NKG2DL on cancer cells, so NKG2DL upregulation may improve gamma delta T cell recognition of GBM cells to improve this cell-based immunotherapy [18].

While the optimal strategy for improving gamma delta T cell recognition and cytotoxicity of GBM cells has not been determined, NKG2D ligands (NKG2DL) for the NKG2D receptor are induced by cell stresses that include DNA damage [16,17]. Chemotherapeutic agents that increase DNA damage induced NKG2DL on a variety of cancer cell types. In GBM, TMZ induced a transient expression of NKG2DL in the U87 cell line and enhanced the cytotoxic efficacy of genetically engineered TMZ-resistant gamma delta T cells [11,12,13]. Although increasing DNA damage to decrease GBM growth is being clinically tested with TMZ and PARP inhibitor treatment (described above), the effects of TMZ and PARP inhibition on NKG2DL expression in GBM have yet to be investigated. We hypothesized that the added cell stress of TMZ and PARP inhibitor treatment would induce NKG2DL on GBM cells. If true, we expected that our studies would suggest a mechanism to improve gamma delta T cell recognition of GBM cells. Utilizing combined treatments of TMZ and the FDA-approved, blood–brain barrier penetrant, dual PARP1/2 inhibitor, niraparib, we found that this drug combination increased NKG2DL on GBM cells derived from multiple patient-derived xenografts (PDX) and increased the killing of GBM39 PDX derived cells by gamma delta T cells. We suggest that there is potential for using chemotherapy-induced upregulation of NKG2DL through the combination of TMZ and PARP inhibition in the appropriate immunotherapy setting for GBM treatment.

## 2. Materials and Methods

### 2.1. Cell Lines

GBM39 and GBM22 patient-derived xenograft cells (PDXs) were gifted by Dr. Jann Sarkaria (Mayo Clinic, Rochester, MN, USA). UAB GBM1016 xenograft cells, U87 cells, and mouse syngeneic SB28 cells were obtained as previously described [19]. The propagation of GBM PDXs was maintained by the establishment of tumors via subcutaneous injection of minced tissue or cells into Balbc nu/nu mice. Tumor implantation and monitoring were in accordance with guidelines set by UAB’s Institutional Animal Care and Use Committee. As we have previously described, once tumors reached a diameter of about 10 mm, xenografts were harvested and dissociated using a Papain Dissociation Kit (Worthington Biochemical Corporation, Lakewood, NJ, USA; LK003150) [20,21]. To maintain molecular features of dissociated PDX, cells were cultured in vitro for no more than 10 passages. Harvested gamma delta T cells were provided by In8Bio and cultured as previously described [11,13].

### 2.2. Cell Culture

Cells isolated from GBM PDXs were maintained in serum-free culture conditions to enrich brain tumor-initiating cells (BTICs) and better recapitulate the heterogeneity associated with GBM. PDX culture media consisted of DMEM/F12 (Gibco/Thermo Fisher Scientific, Waltham, MA, USA; cat. no. 21041025) supplemented with GEM21 NeuroPlex without Vitamin A (Gemini Bioproducts, West Sacramento, CA, USA; cat. no. 400-161), 100 U/mL penicillin and 100 µg/mL streptomycin (Gibco; cat. no. 15140122), 1% sodium pyruvate (Gibco; cat. no. 11360070), and 10 ng/mL of epidermal growth factor and fibroblast growth factor (Gemini Bioproducts; cat. no. 300-110P and 300-112P). Murine SB28 cells were cultured using RPMI 1640 (Gibco; cat. no. 21870076) supplemented with 10% fetal bovine serum (FBS) (Peak Serum, Bradenton, FL, USA; cat no PS-FB2), 1% GlutaMAX (Gibco; cat. no. 35050-061), and penicillin and streptomycin (Gibco; cat. no. 15140122). Ex vivo expanded gamma delta T cells were cultured using RPMI 1640 (Gibco; cat. no. 21870076), 10% FBS (Peak Serum; cat. no. PS-FB2), 1% GlutaMAX (Gibco; cat. no. 35050-061), and 1% sodium pyruvate (Gibco; cat. no. 11360070). While under in vitro culture conditions, gamma deltas T cells were stimulated using 400 IU/mL of IL-2 (R&D Systems, Minneapolis, MN, USA; cat. no. 202IL010CF).

### 2.3. Relative Viability

GBM xenograft and syngeneic cells were seeded in 96 well plates at densities between 2 × 10^4^ to 5 × 10^4^ cells per well. Following overnight recovery, cells were treated with serial dilutions of niraparib (MK-4827) (Selleckchem, Houston, TX, USA; cat. no. S2741) to establish optimal treatment concentrations for a 72 h timeframe. Cell viability was then assessed by measuring changes in ATP using Cell Titer Glo 2.0 (Promega, Madison, WI, USA; cat. no. G9243). For combination treatments, 72 h experiments were performed using single agent concentrations of TMZ (100 µm; Selleckchem; cat. no. S1237), niraparib (5–15 µM), or the combination and assessed for cell viability as described above.

### 2.4. Transcriptional Profiling

Cells were plated in 6-well plates at a density of 3 × 10^5^ cells per well. The following day, cells were exposed to TMZ (100 µM), niraparib (5–15 µM), or the combination for a total exposure of 8 h. Following treatment, RNA was harvested using the RNeasy Mini Kit (Qiagen, Germantown, MD, USA; cat. no. 74106) following the manufacturer’s protocol. cDNA was generated using the 5× iScript cDNA synthesis kit (Bio-Rad, Hercules, CA, USA; cat. no. 1708889), and RT-PCR was performed using the ssoadvanced SYBR green qPCR master mix in accordance with the manufacturer’s settings (Bio-Rad; cat. no. 1725274) with primers purchased from Integrated DNA Technologies, Coralville, Iowa, USA. IDT-individual primer sequences are shown below.
**Human NKG2DL****Forward Primer Sequence****Reverse Primer Sequence***MICA*CCA CCA CGA TTT GCC AAG GAG ACTG CCA ATG ACT CTG AAG CAC C*MICB*GGA ATG GAA CCT ACC AGA CCT GCTG TCC GTT GAC TCT GAA GCA C *ULBP1*CTG CTT GAC ATT CAA GTG GAG AATCTG TCC ATT GAA GAG GAA CTG CC *ULBP2*GAG CAA CTG CGT GAC ATT CAG C GCC CAT CGA AAC TGA ACT GCC A *ULBP3*CAG ACT GGA ACT GGC TGA CACTGGA GGA ACT TCC GTC CAT CGA A*ULBP4*GGT ATA TGC CAC CAG CAC TTG GTCT CGA CTT GCA GAG TGG AAG G*ULBP5*CTG CTG AGA AGA GTC CTT TGG AG GGT AAG GAG TGT GAG TCG TCT C*ULBP6*AGA GCA ACT GCT TGA CAT TCA GCGAG TAG GAA GGT CTG TCC ATC G **Murine NKG2DL****Forward Primer Sequence****Reverse Primer Sequence***Mult-1*GTG CAG GAG ACT AAC ACA ACC G TGC CAG TGC TTG TGT CAA CAC G 

### 2.5. Flow Cytometry

GBM cells were seeded at 1 × 10^6^ cells per 100 mm plate and pretreated for 24 h with the respective vehicle, TMZ, niraparib, or the combination as determined by initial cell viability assays. After pretreatment, cells were washed with PBS and labeled with either ULBP1 (R&D Systems; cat. no. FAB1380P) or MULT1 (R&D Systems; cat. no. FAB2588P) antibodies for 30 min on ice. After staining, cells were washed twice with PBS, resuspended in 250 µL buffer (PBS with 2% FBS), and filtered through a 70 µm filter for flow acquisition using BD FACS Symphony A5 (Ganymede). Data were further visualized and analyzed using FlowJo 10.8.1 where median fluorescent intensity was established for each respective treatment group.

### 2.6. Cytotoxicity

GBM cells (GBM39) were subject to in vitro cytotoxicity assays after 24 h pretreatment with respective concentrations of vehicle, TMZ, niraparib, or the combination. Prior to pretreatment, GBM cells were labeled (1:1000) with the membrane-permeable fluorescent dye CellTracker Green CMFDA (Thermo Fisher; cat. no. C2925). Genetically unmodified gamma delta T cells (isolated and expanded as previously described) were then added to the GBM cells at ratios of 0:1 and 10:1 using a Nunc Sphera 96-well plate with cells seeded in triplicate wells for each treatment group [11,13]. Cocultured cells were then incubated for 4 h at 37 °C and 5% CO_2_. After incubation, cells were collected and stained (1:1000) with Sytox Orange nucleic acid dye (Thermo Fisher; cat. no. S11368), followed by a 30 min incubation on ice. Cells were then washed once and resuspended in 200 µL PBS for flow cytometry analysis.

### 2.7. Statistical Analysis

Graph Pad prism (version 9.3.1) was used for statistical analyses with *p* < 0.05 indicating statistical significance. The statistical tests performed and *p* values for specific comparisons made are detailed in the figure legends.

## 3. Results

### 3.1. Temozolomide and Niraparib Decrease Human and Mouse Glioma Cell Growth

**Figure 1:**
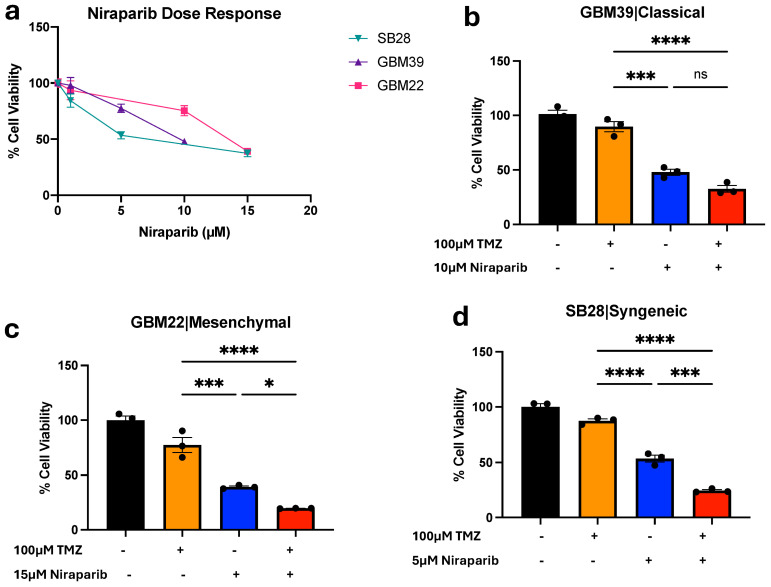
GBM Cell Sensitivity to Combined PARP Inhibition and Temozolomide. (**a**) Dose response inhibition of GBM PDX and syngeneic models to single 72 h treatment with niraparib or vehicle. Respective combinatorial benefit of (**b**) GBM39, (**c**) GBM22, and (**d**) SB28 cells treated with TMZ or niraparib alone or in combination in comparison to vehicle for 72hrs. Relative viability was measured using Cell Titer Glo. Data are displayed as means ± SEM. Not significant (ns), * *p* < 0.05, *** *p* < 0.001, **** *p* < 0.0001 as analyzed by one-way ANOVA.

PARP inhibitors, as well as combinations of PARP inhibitors with TMZ, have been reported to decrease GBM growth [4,22]. We confirmed that niraparib could decrease the growth of GBM cells derived from two human xenografts (GBM39, GBM22) as well as a murine syngeneic glioma model (SB28; Figure 1a). Approximate IC50 concentrations were determined for each cell type to use in combinatorial treatments with TMZ (Figure 1b–d). The combination of TMZ and niraparib significantly decreased cell growth compared with the vehicle control in all models tested (Figure 1b–d). Growth was significantly decreased compared with each single agent in two of the three models (Figure 1b–d). Our findings supported and validated previous studies suggesting the benefit of the combination of TMZ and PARP inhibitors for inhibiting GBM cell growth.

### 3.2. Temozolomide and Niraparib Increase ULBP1/Mult-1 mRNA

**Figure 2:**
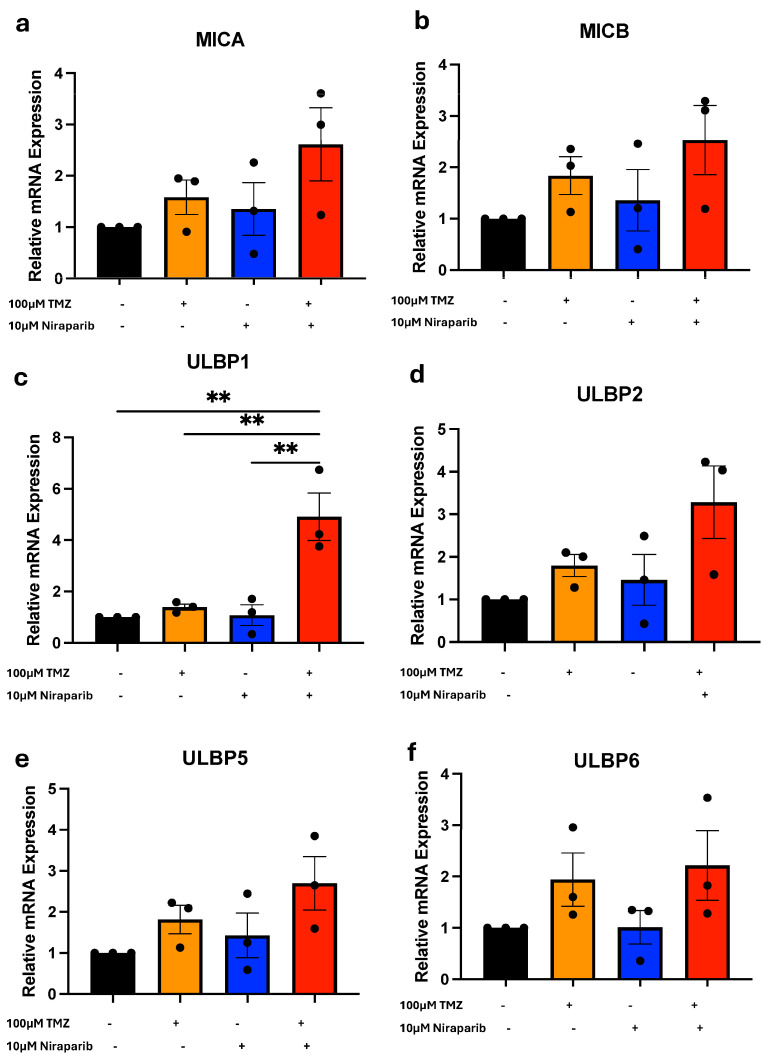
Induction of ULBP1 mRNA with TMZ and niraparib treatment. RT-PCR analysis of the NKG2DLs (**a**) MICA, (**b**) MICB, (**c**) ULBP1, (**d**), ULBP2, (**e**) ULBP5, or (**f**) ULBP6 in GBM39 cells following 8 h exposusre to vehicle, TMZ, or niraparib or the combination of TMZ and niraparib. N = 3 independent experiments performed in duplicate. Data are presented as means ± SEM. ** *p* < 0.01 as analyzed by One-Way ANOVA.

To expand on the known effects of TMZ and PARP inhibitor combinations, we next determined the effects on the expression of known NKG2DL. As NKG2D ligands are transiently expressed and gene expression changes can provide biomarkers for therapeutic response, we first evaluated changes in NKG2DL at the level of transcription after short-term treatments. Following an 8 h exposure to either vehicle, TMZ, niraparib, or the combination, NKG2DL mRNA expression in cells derived from GBM39 [*MICA* (Figure 2a), *MICB* (Figure 2b), *ULBP1* (Figure 2c)*, ULBP2* (Figure 2d), *ULBP5* (Figure 2e), and *ULBP6* (Figure 2f); *ULBP3* and *ULBP4* in Figure S1) or GBM22 (*MICA*, *MICB, ULBP1*, *ULBP2*, *ULBP5*, and *ULBP6* in Figure S2) was determined using real-time PCR. Comparable treatments were performed in SB28 cells, and mRNA expression of the murine NKG2DL *Mult-1* (Figure S2a) was determined. There was a trend toward broad NKG2DL mRNA upregulation in response to the combinatorial treatment as compared with single agents or the vehicle control (Figure 2, Figures S1 and S2). However, the only genes that were significantly altered with TMZ and niraparib treatment in comparison to the vehicle control or either drug alone were *ULBP1* (UL16 binding protein) in GBM39 cells (Figure 2c) or *Mult-1* in SB28 cells (Figure S2a). While the GBM22 cells were stressed by the combination of DNA alkylation and PARP inhibition, as evidenced by the resulting growth inhibition, there was no statistically significant benefit for the combination to induce NKG2DL at the mRNA level (Figure S2).

### 3.3. Temozolomide and Niraparib Increase ULBP1/Mult-1 Protein

**Figure 3:**
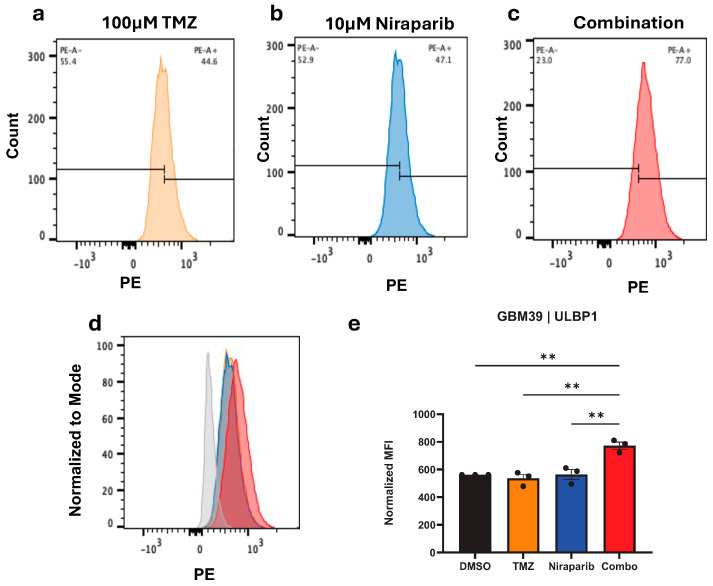
ULBP1 protein is increased by TMZ and niraparib. GBM 39 cells were pretreated for 24 h with respective single agent concentrations of (**a**) TMZ or (**b**) niraparib or (**c**) the combination and then labeled with ULBP1-PE conjugated antibody for flow acquisition. (**d**) Overlayed data plots and (**e**) normalized MFI are displayed. Data are presented as means ± SEM. ** *p* < 0.01 as analyzed by One-Way ANOVA.

**Figure 4:**
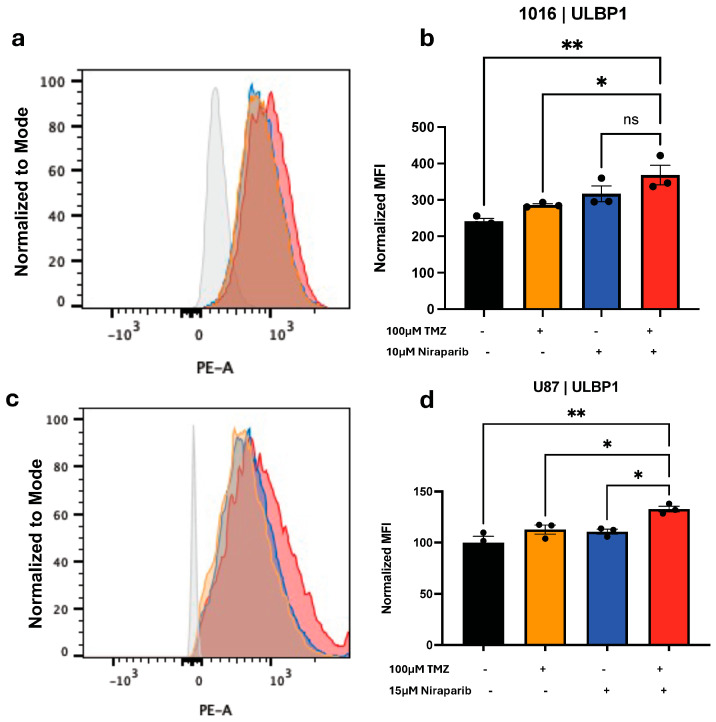
ULBP1 Protein Significantly Increases with TMZ and Niraparib Treatment of GBM1016 and U87 GBM Cells. (**a**,**b**) PDX derived 1016 or (**c**,**d**) U87 cells were pretreated for 24 h with the vehicle, TMZ, niraparib, or the combination of TMZ and niraparib and then labeled with ULBP1-PE conjugated antibody for flow acquisition. (**a**,**c**) Overlayed histograms and (**b**,**d**) normalized MFI are displayed. Data are presented as means ± SEM. Not significant (ns), * *p* < 0.05 ** *p* < 0.01 as analyzed by one-way ANOVA.

Based on the change in NKG2DL mRNA levels, the next step is to determine if the combination of TMZ and niraparib increased levels of ULBP1 or MULT-1 protein. Glioma cells were exposed to either a single agent or a combination of TMZ and niraparib for 24 h. Following treatment, cells were labeled with fluorescently conjugated antibodies against ULBP1 (Figure 3 and Figure 4) or MULT-1 (Figure S3b–f), and cell surface protein levels were determined via flow cytometry. We determined that expression of ULBP1 protein in the human GBM39 (Figure 3), GBM1016 (Figure 4a,b), and U87 (Figure 4c,d) cells was significantly increased in comparison to either drug alone (Figure 3 and Figure 4c,d) or the vehicle control (Figure 4a,b). However, there was no change in ULBP1 cell surface protein levels in GBM22 cells with any treatment, which was consistent with the lack of mRNA induction (Figure S2). In the murine glioma cells, the highest level of MULT-1 cell surface protein was observed in cells treated with the combination of TMZ and niraparib (Figure S3b–f). The level of this NKG2DL was significantly higher in the combination when compared with either TMZ or niraparib alone (Figure S3b–f). To make these comparisons, we used unstained cells as a representative control (grey peak on histogram; Figure 3d, Figure 4a,c, Figures S3e and S4a) and compared the normalized median fluorescence intensity of ULBP1 (Figure 3e, Figure 4b,d and Figure S4b) or MULT1 (Figure S3f). Together, these data indicate that the combination of TMZ and PARP inhibitors can induce NKG2DL in the majority of glioma cells tested, in addition to directly decreasing cell growth.

### 3.4. Pretreatment with Temozolomide and Niraparib Increases the Ability of Gamma Delta T Cells to Kill GBM39 Cells

**Figure 5:**
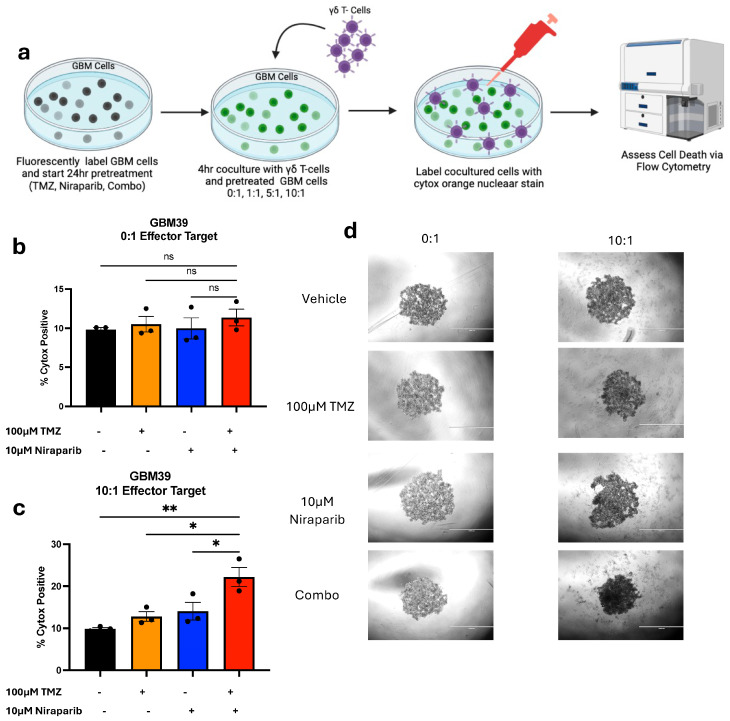
TMZ and niraparib pretreatment enhances the cytotoxicity of gamma delta T cells. (**a**) Representative schematic of the experimental layout for vehicle, TMZ, niraparib, or TMZ and niraparib pretreated GBM39 cells co-cultured with gamma-delta T cells. Respective GBM39 cells (**b**) without and (**c**) with a 10:1 ratio of gamma delta T cells were analyzed for percentage of sytox positive cells following 4 h co-coculture. (**d**) Representative images for the treatment groups are displayed. Data are presented as means ± SEM. Not significant (ns), * *p* < 0.05 ** *p* < 0.01 as analyzed by One-Way ANOVA.

As NKG2DL upregulation at the protein level was validated, we further investigated whether TMZ and niraparib pre-treatment would enhance the cytotoxic killing of gamma delta T cells (schematic Figure 5a). For this experiment, fluorescently labeled GBM39 cells were subject to a 24 h pretreatment with TMZ, niraparib, or the combination. The fluorescently labeled pretreated cells were then cultured without (Figure 5b) or with (Figure 5c) gamma delta T cells that were stimulated with IL-2. Gamma delta T cells were used at a 10:1 effector-to-target ratio (Figure 5), as previously published research by Lamb et al., demonstrated a 10:1 effector-to-target ratio was optimal for GBM killing post-TMZ treatment [13]. After a 4 h incubation, cells were visualized via light microscopy (Figure 5d) and then collected. Cells were labeled with the viability dye SYTOX orange, and cell death was assessed via flow cytometry (Figure 5b,c). We determined that pretreatment with the combination of TMZ and niraparib enhanced gamma delta T cell-mediated GBM cell death significantly more than either the drug alone or the vehicle control. Overall, these data suggest a potential benefit for combining DNA alkylation and PARP inhibition to improve gamma delta T cell immunotherapy.

## 4. Discussion

### 4.1. Overview of Findings and Clinical Implications

Due to their location and physiologic and molecular complexity, GBMs have proven to be extremely difficult to target therapeutically. Adoptive cell immunotherapy has shown extraordinary promise in a variety of other malignancies, warranting further investigation and optimization of this treatment strategy for GBM [15,23,24,25]. Our study was aimed at determining the in vitro efficacy of dually inducing DNA damage and inhibiting PARP repair mechanisms to promote cellular stress ligands. Our findings validated previous reports that demonstrated TMZ and PARP inhibition decrease GBM growth. Importantly, we further determined that this therapeutic combination can enhance the NKG2DL ULBP1 in vitro in multiple GBM PDXs. This completely novel finding was complemented by proof of concept studies demonstrating TMZ and PARP inhibitor pretreatment increased immune cell-mediated cytotoxicity in cells derived from a GBM PDX, which had not been previously demonstrated. These results in human cells were complemented by results in mouse syngeneic SB28 glioma cells, which model the immunosuppressive characteristics of glioblastoma as they fail to respond to immunotherapies [19,26]. Considering that the induction of ULBP1 was mediated by short-term treatment with TMZ and niraparib and combinations of DNA-damaging agents and PARP inhibitors are in clinical trials, these studies may have important implications for patient treatments. For example, the benefit of drug combinations that increase antigen presence for immune cell recognition may be minimized by immunosuppression caused by the same chemotherapies. Changing the treatment course to add gamma delta T cells when NKG2DL are elevated could optimize the ability to target the GBM and minimize side effects. Existing TMZ-resistant gamma delta T cells [genetically modified to express MGMT] could also be used with continued TMZ treatment after a short-term niraparib treatment. Further characterization of immunosuppressive responses may permit targeting with immune checkpoint inhibitors, thereby increasing gamma delta T cell efficacy. Thus, the combination of TMZ and PARP inhibition could provide alternative ways to target historically immunologically “cold” tumors.

### 4.2. NKG2DL Regulation Including Specific Mechanisms for ULBP1 Induction Need Additional Investigation

There are several NKG2DL that commonly bind and activate the NKG2D receptor, but the differences in the function and regulation of these ligands have yet to be fully understood. With regards to their regulation, NKG2DL transcription can be changed by miRs and kinase signaling. For example, miR-20a, miR-93, and miR-93 suppress NKG2DL to promote immune escape in GBM [27]. Cell signaling cascades involving the activation of ATM/ATR kinases, as well as DNA damage sensory pathways, have been directly linked to NKG2DL mRNA expression [28]. As we observed congruence between ULBP1 mRNA and protein expression, we expect that the activity of NKG2DL regulating pathways is altered upon treatment with TMZ and niraparib in GBM to upregulate *ULBP1* and the resulting protein expression. We, therefore, anticipate that it could be informative to perform RNA-seq on GBM cells treated with a time course of TMZ and niraparib in comparison to single agent and vehicle controls. Pathways induced prior to *ULBP1* could be important regulators of its transcription, and it would be beneficial to know if these pathways were specifically inducing *ULBP1* versus other NKG2DL. However, it is important to note that NKG2DL are also regulated at the post-transcriptional level: ubiquitination can decrease in response to stress, leading to reduced degradation and increased cell surface expression [28]. Thus, further investigation is needed to confirm the exact mechanisms through which ULBP1 is upregulated after TMZ and PARP inhibition.

### 4.3. ULBP1 Induction Can Be Distinct from Cell Stress Indicated by Decreased GBM Cell Growth

Although all GBM cell types tested had decreased growth with the combination of TMZ and niraparib compared with the vehicle control, one did not have increased induction of any NKG2DL, including ULBP1. This indicates that decreased GBM cell growth cannot be a marker of cell stress-mediated induction of NKG2DL and that there are likely to be subsets of GBM cells that will not induce NKG2DL in response to TMZ and PARP inhibitor treatment. Whether NKG2DL mRNA levels or cell surface expression post-TMZ and niraparib treatment could predict gamma delta T cell-based immunotherapy efficacy, therefore, remains an important area of investigation. When exploring this possibility, it will be important to consider if intratumoral heterogeneity could explain the varied responses in NKG2DL expression using this targeted approach. The GBM39 and GBM22 PDX models represent two of the molecular subtypes of GBM, classical and mesenchymal, respectively, which are present in all patients [29]. The mesenchymal subtype of glioblastoma is more difficult to target with standard therapies, corresponding to poorer overall prognosis [30,31]. The mesenchymal subtype is also characterized by greater immune infiltration of pro-tumorigenic macrophage and microglia cells (TAMs), which may further contribute to an immunosuppressive tumor microenvironment: this could negate the potential benefit of NKG2DL induction in vivo [32]. Further elucidating NKG2DL regulation between GBM cell types to understand whether there are differential NKG2DL expression patterns when using this targeted approach is, therefore, an important area for investigation.

### 4.4. Limitations of the Study and Future Directions

Since clinical trials with TMZ and PARP inhibitors are ongoing and gamma delta T cells are already being injected into the brains of glioblastoma patients through Rickham catheters, we believe that this study has translational potential. However, we recognize that there are multiple limitations and that several important preclinical experiments are needed to further confirm whether the combination of TMZ, niraparib, and gamma delta T cells is likely to be beneficial for GBM patients. The first challenge is to establish the time course over which ULBP1 expression is induced and maintained in GBM cells in vivo. Our in vitro studies were based, in part, on a prior report by Lamb et al., that provided a time course for multiple NKG2DLs to be induced post-TMZ treatment [11,13] and demonstrated peak expression of ULBP1 at 8 h with elevated expression maintained for 24 h. We similarly observed elevated ULBP1 elevation with TMZ and niraparib treatment at 24 h post-treatment in vitro, but it is unclear whether this induction would be observed or observed with a similar time course in vivo. Thus, it will be important in future studies to use flow cytometry or immunohistochemistry with antibodies to ULBP1 to analyze the brains of mice bearing different GBM PDXs that are treated with vehicle, single agents, or TMZ and niraparib via oral gavage and sacrificed at increasing amounts of time post-treatment. Once the time at which TMZ and niraparib induce ULBP1 in vivo is known, preclinical studies, including the addition of gamma delta T cells, could be planned. Preferably, a time at which ULBP1 is induced when TMZ and niraparib are eliminated (to avoid toxicity to the immune cells) would be defined. As it may be important to predict how many gamma delta T cells are required for efficacy, additional studies with multiple ratios of effector to target cells could be performed. To predict whether increased gamma delta T cell cytotoxicity against GBM cells is broadly applicable, it would also be important to demonstrate the ability of gamma delta T cells to kill cells derived from multiple GBM PDXs: we only demonstrated increased cytotoxicity in one cell type in proof of concept studies. Once these studies were completed, preclinical trials could determine the ability of TMZ and niraparib treatment with gamma delta T cell intracranial injection to increase the survival of mice bearing orthotopic gliomas.

## 5. Conclusions

The NKG2DL ULBP1 was increased by a clinically utilized treatment of TMZ and PARP inhibition. As NKG2DLs on tumor cells bind NKG2D to promote immune cell recognition, the indirect benefits of DNA alkylating agent and PARP inhibitor combinatorial therapy are likely relevant for cell-based immunotherapies. Indeed, TMZ and PARP inhibitor treatment of cells derived from a GBM PDX increased gamma delta T cell killing. As the elevation of ULBP1 mRNA correlated with increased protein levels, *ULBP1* induction should be further explored as a biomarker for response to cell-based immunotherapies in GBM. Finally, the potential benefit of combining TMZ, niraparib, and gamma delta T cell therapy for glioma treatment should be further elucidated in preclinical trials.

## Data Availability

Data is available upon request.

## Supplementary Materials

The following supporting information can be downloaded at: https://www.mdpi.com/article/10.3390/cancers16162852/s1, Figure S1: No Changes in Gene Expression of ULBP3 and ULBP4; Figure S2: TMZ and Niraparib Do Not Significantly Induce NKG2DL Gene Expression in GBM22; Figure S3: MULT-1 Protein Induction in SB29 Syngeneic Cells; Figure S4: ULBP1 Protein Does Not Increase with TMZ and Niraparib Treatment in GBM22 Cells.
