# Supplementary material for: Temozolomide and the PARP Inhibitor Niraparib Enhance Expression of Natural Killer Group 2D Ligand ULBP1 and Gamma-Delta T Cell Cytotoxicity in Glioblastoma
Source: Cancers (Basel). 2024 Aug 15;16(16):2852. doi: 10.3390/cancers16162852 (PMC11352900; doi:10.3390/cancers16162852)
Supplement: Supplementary file 1 [file cancers-16-02852-s001.zip › cancers-3088907-supplementary.pdf]

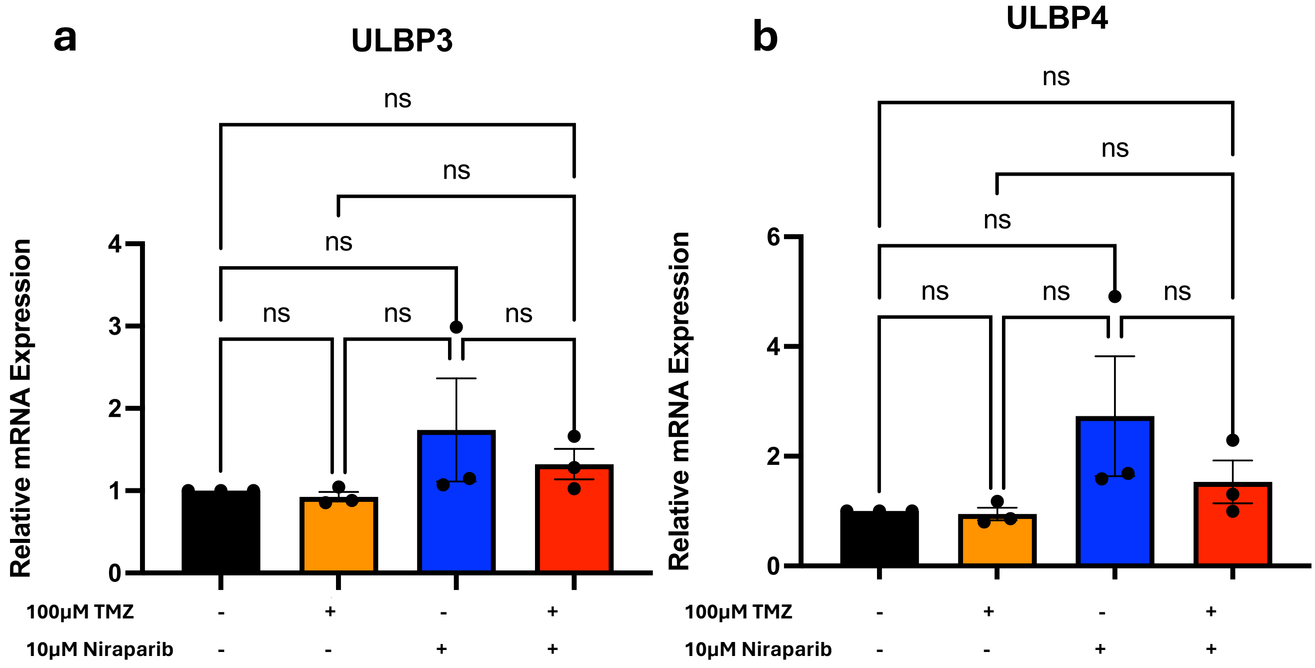

Supplemental Figure S1. No Changes in Gene Expression of ULBP3 and ULBP4. RT-PCR analysis of various (**a**) ULBP3 or (**b**) ULBP4 in GBM39 cells following 8 h exposure either singular treatment of TMZ or niraparib or the combination. N = 3 independent experiments performed in duplicate. Data are presented as means ± SEM. Not significant (ns).

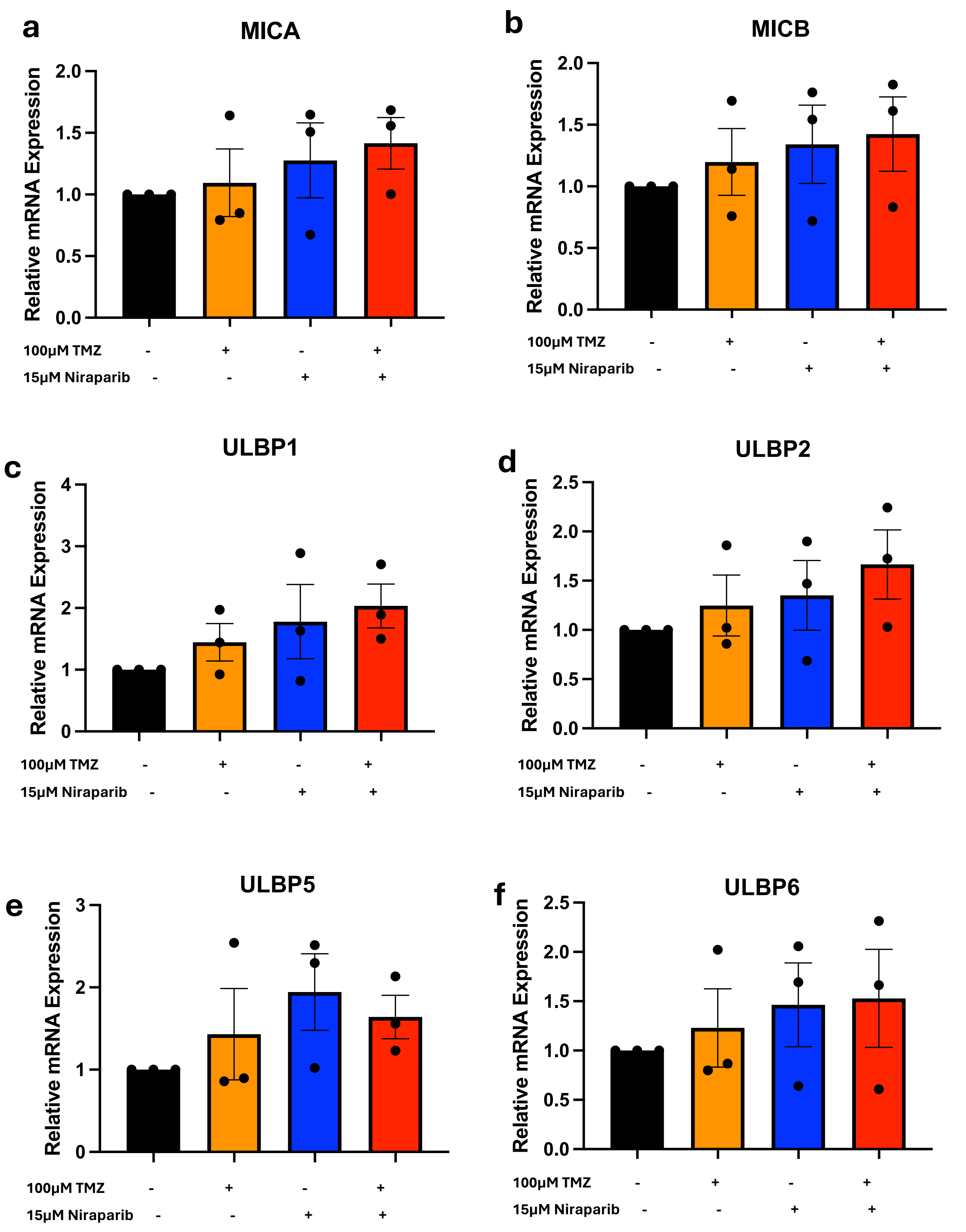

Supplemental Figure S2. TMZ and Niraparib Do Not Significantly Induce NKG2DL Gene Expression in GBM22. RT-PCR analysis of the NKG2DLs (**a**) MICA, (**b**) MICB, (**c**) ULBP1, (**d**) ULBP2, (**e**) ULBP5, or (**f**) ULBP6 in GBM22 cells following 8hr exposure to vehicle, TMZ, or niraparib or the combination of TMZ and niraparib. N = 3 independent experiments performed with duplicates. Data are presented as means ± SEM.

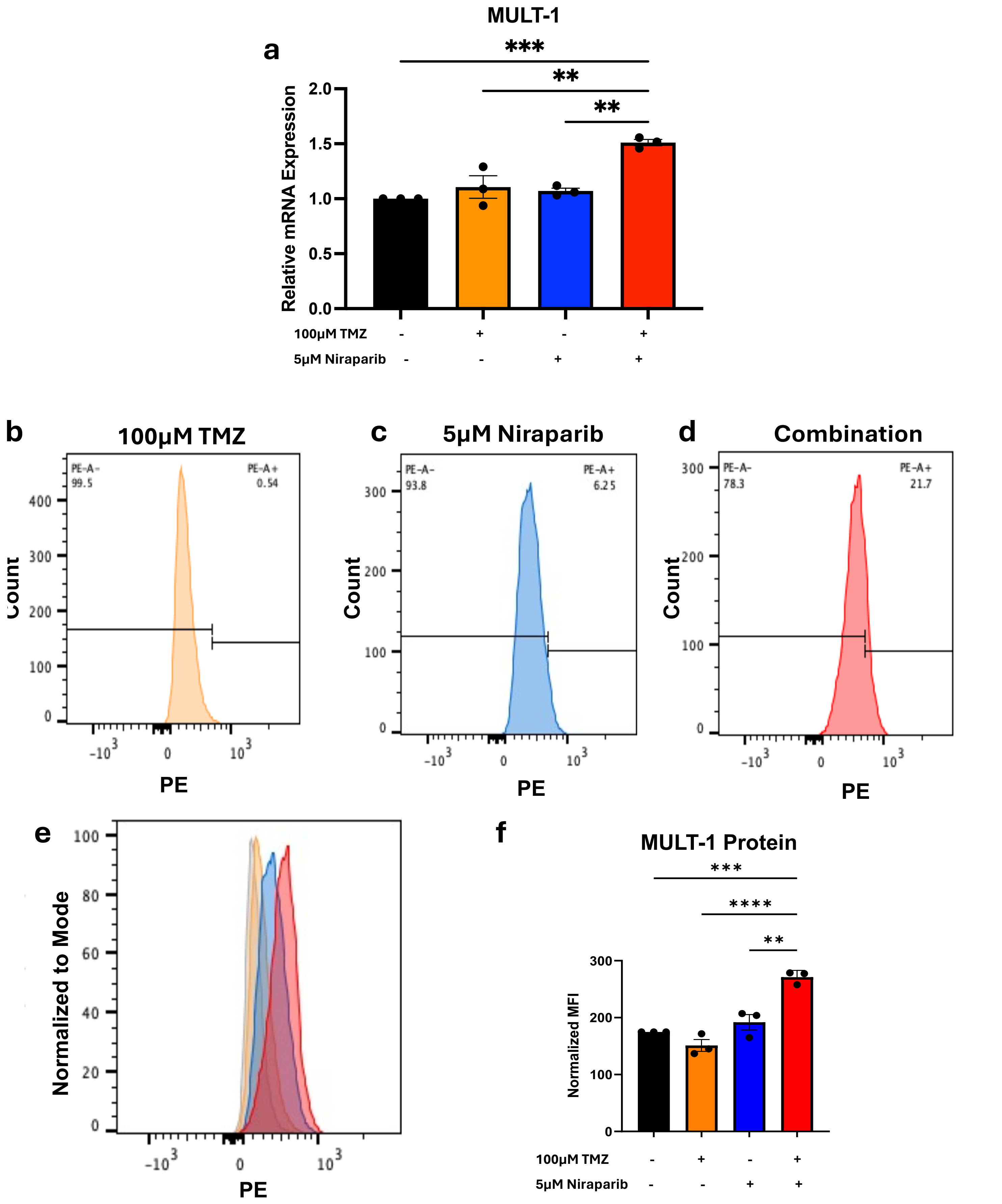

Supplemental Figure S 3. MULT-1 Protein Induction in SB29 Syngeneic Cells. (**a**) RT-PCR analysis of various MULT-1 in SB28 cells following 8hr exposure to either vehicle, TMZ, niraparib, or TMZ and niraparib in combination. Cells were pretreated for 24 hours with respective concentrations of (**b**) TMZ, (**c**) niraparib or (**d**) TMZ and niraparib in combination and then labeled with ULBP1-PE conjugated antibody for flow acquisition. (**e**) Overlayed histograms including the DMSO vehicle control and (**f**) normalized MFI are displayed. For (**b**–**e**), representative histograms are shown. For (**a**) and (**f**), data are presented as means ± SEM from N = 3 independent experiments. Not significant (ns), ** $p < 0.01$, ***, $p < 0.001$, as analyzed by one-way ANOVA.

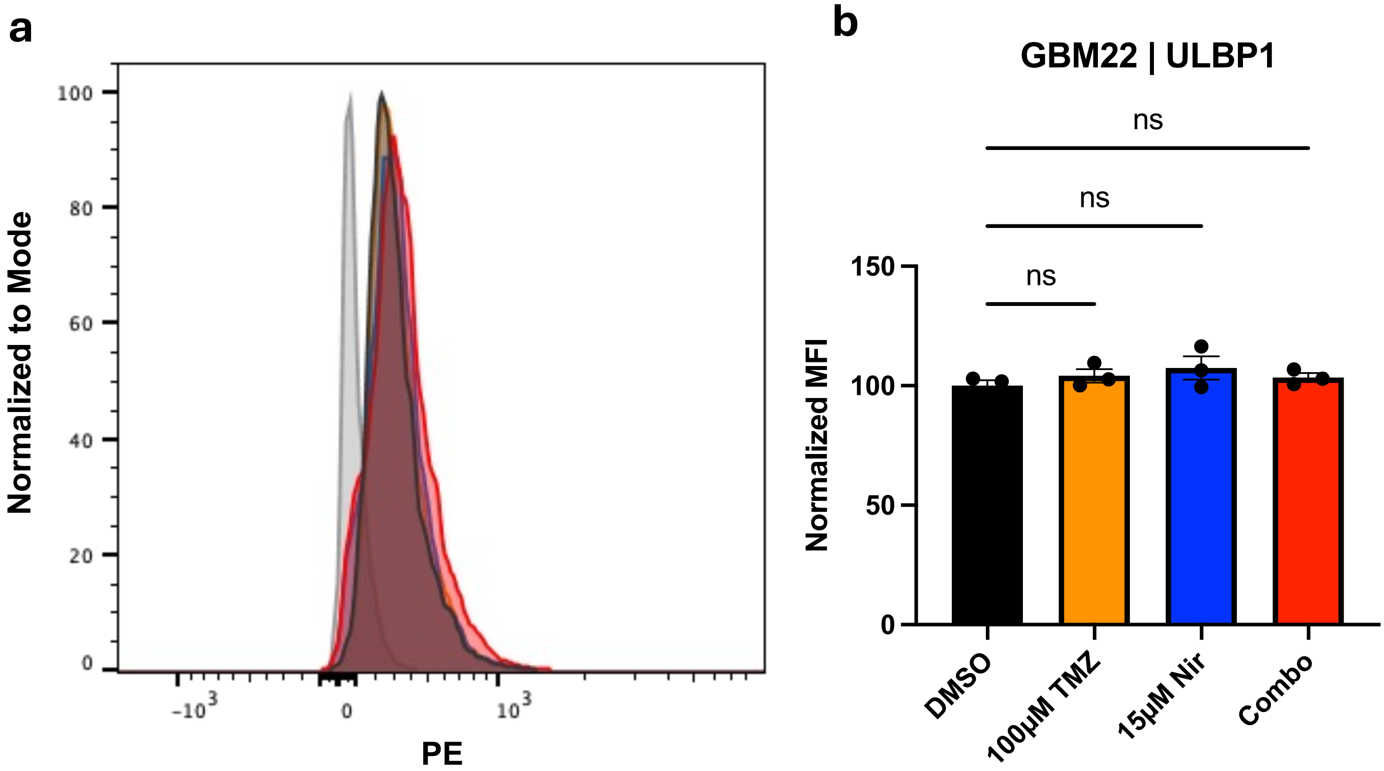

Supplemental Figure S 4. ULBP1 Protein Does Not Increase with TMZ and Niraparib Treatment in GBM22 Cells. Cells were pretreated for 24 hours with the vehicle, TMZ, niraparib, or the combination of TMZ and niraparib and then labeled with ULBP1-PE conjugated antibody for flow acquisition. (**a**) Overlayed histograms from a representative experiment are shown. (**b**) Normalized MFI are displayed as means ± SEM from N = 3 independent experiments. Not significant (ns).
